# Significant reduction of long non-coding RNAs expression in bipolar disorder

**DOI:** 10.1186/s12888-022-03899-y

**Published:** 2022-04-12

**Authors:** Zahra Maloum, Mohammad Taheri, Soudeh Ghafouri-Fard, Zeinab Shirvani-Farsani

**Affiliations:** 1 Department of Cell and Molecular Biology, Faculty of Life Sciences and Technology, Shahid Beheshti University, Tehran, Iran; 2 Skull Base Research Center, Loghman Hakim Hospital, Shahid Beheshti University of Medical Sciences, Tehran, Iran; 3 Department of Medical Genetics, School of Medicine, Shahid Beheshti University of Medical Sciences, Tehran, Iran

**Keywords:** RMST lncRNA, MEG3, Stress oxidative, Bipolar, Biomarker, Brain

## Abstract

Long non-coding RNAs (lncRNAs) have been recently emerged as critical modulators of oxidative stress pathway. Likewise, rising evidence currently highlights dysfunction of oxidative stress pathways in bipolar disorder (BD) patients.

In the current study, we evaluated the expression levels of *H19*, *SCAL1* (*LUCAT1*), *RMST*, *MEG3* and *MT1DP* lncRNAs in the PBMC from 50 patients with BD and 50 control subjects (male/female ratio in each group: 70%/30%). Expression levels of *SCAL1*, *RMST* and *MEG3* but not *H19* and *MT1DP* were considerably decreased in BD patients compared with healthy individuals. Such significant decrease in the expression of *MEG3*, *RMST* and *SCAL1* was only reported in male BD patients compared with male controls. Substantial pairwise correlations were observed between expression levels of these lncRNAs in BD subjects. The area under curve values for *RMST*, *MEG3* and *SCAL1* were 0.70, 0.63 and 0.61 respectively. On the basis of this finding, *RMST* had the best efficiency in the discrimination of disease status between BD patients and controls. Taken together, the current results suggest a role for *MEG3*, *RMST* and *SCAL1* lncRNAs in the pathogenesis of BD. In addition, peripheral expression levels of these lncRNAs might serve as potential peripheral markers for BD.

## Introduction

Bipolar disorder (BP) is a prevalent psychiatric disorder [1]. Genetics and environmental parameters have roles in the etiology of this disorder. Although researchers have not yet understood how genetics and environment interact with each other to cause this disorder, there are several studies that have reported dysregulated expression of genes in association with the pathogenesis of BD [2–4]. Oxidative stress that triggers cell death is one of the pathways implicated in this disorder. The brain is susceptive to oxidative stress, and it is essential to maintain a redox state for normal function of brain [5]. Long noncoding RNAs (lncRNA) are a kind of regulatory RNAs that modulate the expression of several genes in the oxidative stress pathway [6–8]. These RNAs contain more than 200 nucleotides and critically partake in transcriptional and post-transcriptional modulation of gene expression. They can regulate various biological pathways, including cell differentiation, apoptosis, cell growth, oxidative stress, and many more [9]. Several lines of evidence have indicated dysregulation of lncRNAs in brain tissues and the peripheral blood of patients with BD [2, 10, 11]. Yet, the function of many other lncRNAs in the etiology of this mental illness has not been investigated. In the present research project, we analyzed the expression of five lncRNAs namely *H19*, *SCAL1* (*LUCAT1*), *RMST*, *MEG3* and *MT1DP* in peripheral blood of cases with BD and healthy controls. H19 is one of the lncRNAs that regulate cell stress and harmonize cell fate and differentiation [12]. Research has shown that *H19* is expressed in hippocampal neurons and promotes apoptosis in these neurons [12]. Moreover, *H19* has been found to decrease defects of dopaminergic neurons through modulation of Wnt/β-catenin signaling [13], a pathway which is dysregulated in mood disorders and has a critical role in the development of these conditions [14]. *SCAL1* (*LUCAT1*) is an lncRNA whose knockdown has decreased cell proliferation while inducing apoptosis and cellular stress [15, 16]. This lncRNA has been suggested to be an essential intermediate molecule in the process of regulation of antioxidant molecules by Nrf2 [17]. Nrf2 partakes in the pathogenesis of mood disorders. Knock-out this gene has resulted in induction of depression-like symptoms in the mice in association with increased concentrations of pro-inflammatory cytokines in the serum while decreased levels of BDNF in the prefrontal cortex and hippocampus of animals [18]. *RMST* as an lncRNA that has a role in neuronal differentiation through interaction with SOX2 [19]. Moreover, *RMST* expression has been found to be enhanced during differentiation of neural crest cells. This lncRNA has been suggested as a regulator of GnRH neurons [20]. Overexpression of *MEG3* lncRNA has decreased neuronal activity and increased cell apoptosis [21]. *MEG3* regulates hypoxia-mediated neuron apoptosis through affecting lipoxygenase signaling [22], a pathway which is found to be disturbed in the brain tissues of BD patients [23]. *RMST* and *MEG3* lncRNAs contribute in controlling oxidative stress [24]. LncRNA *MT1DP* protects cells against oxidative stress, particularly the toxicity of cadmium to hepatocytes [25]. Consequently, aberrant expression of these lncRNAs may partake in the pathogenesis of BD or might be used as biomarker of this disease.

## Materials and methods

### Subjects

The Ethical Committee of Shahid Beheshti University of Sciences has approved this project. All participants in this study signed the written informed consent and the study protocol was performed in accordance with the ethical guidelines. Blood specimens of 50 patients and 50 healthy people were collected from Farshchian and Imam Hussein hospitals during 2016–2019. Patients were diagnosed assessed based on the presence of manic and depressive episodes according to the Diagnostic and Statistical Manual of Mental Disorders-5 [26]. All subjects were assessed using a semi-structured interview by experienced psychiatrists. Disease duration, disease onset and medication history were obtained. All BD patients were under treatment with standard dose of Carbamazepine. With the intention of decreasing the heterogeneity of patients’ cohort, patients who were under treatment with other drugs were omitted from the study. None of the patients and controls had a background of head trauma, encephalitis or other mental illnesses, neurodegenerative diseases, cancer, Epstein–Barr virus infection, or other infections.

### Sample collection and RNA extraction

Four ml of peripheral blood was gathered from all people in EDTA tubes. The blood samples were centrifuged at 3000 rpm for 10 min and the Buffy coat was separated. Total RNA was drawn out from the PBMC using RNX kit (EX6101, Cinnagen, Tehran, Iran) according to the manufacturer’s guidelines. Qualitative and quantitative analyses of extracted RNA were performed by gel electrophoresis and the spectrophotometer.

### cDNA production and real-time PCR assay

cDNA was produced using 3 μg of purified total RNA and High-Capacity cDNA Reverse Transcription Kits (Applied Biosystems, PN: 4375575), based on the manufacturer’s rules. The expression levels of lncRNAs were measured in comparison with *GAPDH* as an internal control using appropriate primers (Table 1). Quantitative Real-time PCR was executed in the ABI 7500 sequence detection system (Applied Biosystem, Foster City, CA, USA) using 10 μl of BIOFACT™ 2X Real-Time PCR Master Mix, 10 ng cDNA and 200 nM of each primer. All experiments were performed at least in duplicate. Means of ΔCT for cases and controls were calculated and, lastly, the fold changes of expressions of genes were measured by ratio = 2^-ΔΔCt^ as explained by Livak [27].

**Table 1:** Primers used in RT-qPCR

Gene	Forward Primer	Reverse Primer	Product Size (bp)
*H19*	5′-TGCTGCACTTTACAACCACTG-3′	5′-ATGGTGTCTTTGATGTTGGGC-3′	105
*MEG3*	5′-TGGCATAGAGGAGGTGAT-3′	5′-GGAGTGCTGTTGGAGAATA-3′	111
*RMST*	5′-CAGGATGGCAGTGGGTGA-3′	5′-GTCCCTTGTGATCTCTGTGAC-3′	137
*SCAL1*	5′-CCCAATGAAAAGGAACAAAACC-3’	5′-ATTTGTGAGGGGATGAGAATAC-3’	217
*MT1DP*	5′-CAAGAAGAACTGCTGCTCCT-3’	5′-TTGTAGGGGTTGCGTTATTTAC-3’	147
*GAPDH*	5′-CCATGAGAAGTATGACAAC-3’	5′-GAGTCCTTCCACGATACC-3’	105

### Statistical method

Statistical analysis was accomplished in the GraphPad Prism 8 (GraphPad Software, Inc., San Diego, CA, USA). Kolmogorov–Smirnov test was used for assessment of normality of distribution of data. The t-test was employed to compare the differences in expression amounts of lncRNAs between patients and controls. Pearson’s coefficient of correlation was measured for appraisal of correlation between lncRNAs expression and the clinical features. *P*-value < 0.05 was considered as significant. Receiver Operating Characteristic (ROC) curve was used to evaluate the specificity and sensitivity of the genes selected as potential biomarkers.

## Results

### Cases and controls

In this study, 50 BD type I patients and 50 healthy individuals were enrolled. General data of patients and healthy controls are presented in Table 2.

**Table 2:** Demographic and clinical features of BD patients and controls

	Cases	Controls
Number	50	50
Sex		
Male (%)	70	70
Female (%)	30	30
Mean age (range)	36 (17–56)	34 (14–52)
Disease duration (range)	4 (1–14)	–
Onset age (range)	32 (15–48)	–

### Gene expression levels in participants

**Fig. 1:**
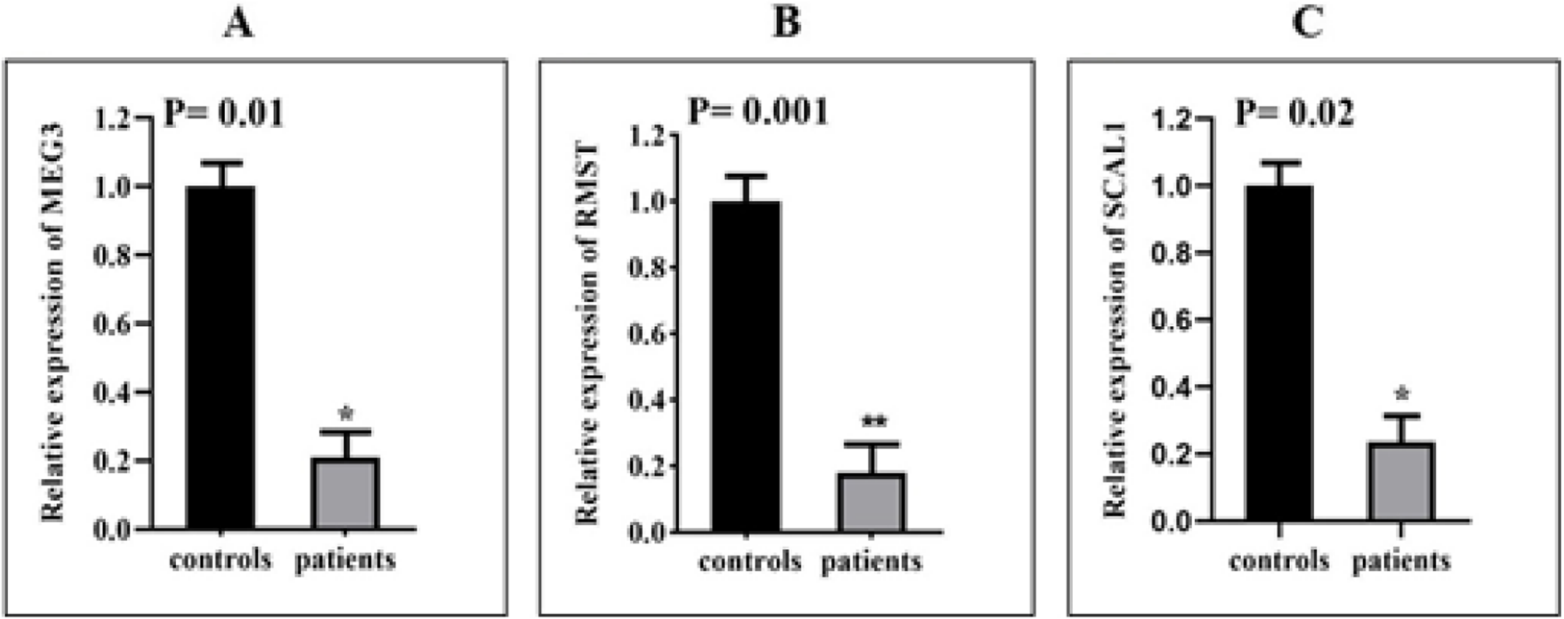
Expression analysis of lncRNAs in the PBMCs. Relative expression (Fold change) of *MEG3* (**A**), *RMST* (**B**), and *SCAL1* (**C**). The expression of lncRNAs was down-regulated in BD patients compared with controls. Expression levels of lncRNAs in each sample were normalized to GAPDH expression. The relative expression of lncRNAs was obtained using the formula 2 ^-ΔΔCt^ and t-test

The expression levels of lncRNAs *MEG3* (*P* = 0.0115), *RMST* (*P* = 0.0013), and *SCAL1* (*P* = 0.0221) were significantly different between BD patients and healthy individuals (Fig. 1 A-C), although, there was no significant difference in the expression levels of lncRNAs *H19* and *MT1DP* between controls and patients (Table 3). In fact, results demonstrated that the expression level of *MEG3*, *RMST*, and *SCAL1* in BD patients decreased 4.76, 5.50, and 4.34 times compared to controls respectively. Furthermore, expressions of lncRNAs *MEG3* (*P* = 0.009), *RMST* (*P* = 0.009), and *SCAL1* (*P* = 0.048) were different between male BD patients and male controls. Instead, there was no important difference in expression of these lncRNAs in female patients compared to control females except for the lncRNA *RMST* (*P* = 0.04). After correction for multiple comparisons, the obtained *P* values remained significant for *RMST* expression in total patients compared with total controls (*P* = 0.005) and in male patients compared with male controls (*P* = 0.005). Moreover, expression of *MEG3* in male patients compared with male controls remained significant (*P* = 0.005). Table 3 displays the outlines of relative expression (fold change) analysis of lncRNAs in BD patients and healthy controls.

**Table 3:** Relative expression of lncRNAs in BD patients and healthy controls

lncRNAs	Parameters	Total patients (*n* = 50)/ total controls (*n* = 50)	Male patients (*n* = 35)/ male controls (*n* = 35)	Female patients (*n* = 15)/ female controls (*n* = 15)
*MEG3*	1/Fold change	4.76	7.69	3.33
	*P*-value	0.011*	0.009**	0.35
*RMST*	1/Fold change	5.50	6.25	12.50
	*P*-value	0.001**	0.009**	0.04*
*SCAL1*	1/Fold change	4.34	5.00	4
	*P*-value	0.022*	0.048*	0.36
*H19*	1/Fold change	1.08	1.33	1.31
	*P*-value	0.90	0.84	0.75
*MT1DP*	1/Fold change	1.12	1.16	1.31
	*P*-value	0.83	0.95	0.94

### Correlation analysis

Significant positive correlations were observed between expression amounts of all pairs of lncRNAs. Table 4 displays the results of pairwise correlation analysis between expression levels of genes. There was no noteworthy correlation between the level of expressions of genes in BD patients and age, disease duration, and onset age of disease (Table 5).

**Table 4:** Pairwise correlation between expression levels of lncRNAs in cases group

Correlation	***MT1DP***	***H19***	***RMST***	***MEG3***
***SCAL1***	*P* < 0.0001	*P* < 0.0001	*P* < 0.0001	*P* < 0.0001
	*r* = 0.8517	*r =* 0.6760	*r =* 0.865	*r =* 0.8811
***MEG3***	*P* < 0.0001	*P* < 0.0001	*P* < 0.0001	
	*r =* 0.8574	*r =* 0.7316	*r =* 0.8296	
***RMST***	*P* < 0.0001	*P* < 0.0001		
	*r =* 0.9107	*r =* 0.7493		
***H19***	*P* < 0.0001			
	*r =* 0.7997

**Table 5:** Appraisal of correlation between expression levels of lncRNAs and clinical data (R is the correlation coefficient or the Pearson’s correlation which measures the closeness of association of the points in the scatter plots to the linear regression line)

	*MEG3*		*RMST*		*SCAL1*		*MT1DP*		*H19*	
	R	***P*** value	R	***P*** value	R	***P*** value	R	***P*** value	R	***P*** value
Patient Age	0.087	0.551	0.148	0.309	0.215	0.136	0.097	0.504	0.041	0.776
Age at onset	0.200	0.166	0.111	0.447	0.191	0.188	0.156	0.284	0.142	0.330
Disease duration	0.160	0.270	0.204	0.158	0.277	0.053	0.190	0.189	0.180	0.215

### ROC curve analysis

**Fig. 2:**
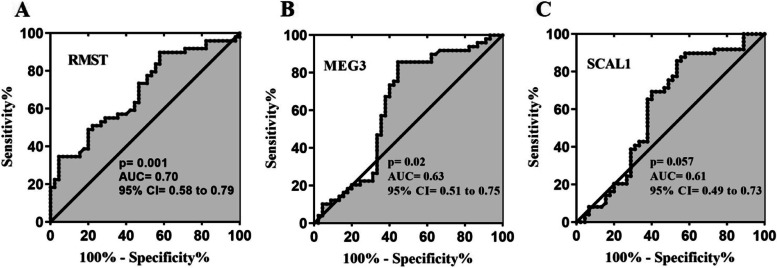
ROC curve analysis of *RMST* (**A**), *MEG3*, (**B**) and *SCAL1* (**C**). AUC: area under curve

The sensitivity and specificity of expression levels of lncRNAs *MEG3*, *SCAL1*, and *RMST* as biomarkers were evaluated using the ROC curve analysis. The results showed that lncRNA *RMST* (AUC = 0.70, Sensitivity = 74%, Specificity = 63%, *P* = 0.001) and lncRNA *MEG3* (AUC = 0.63, Sensitivity = 73%, Specificity = 60%, *P* = 0.028) could differentiate between patients and controls (Fig. 2 A, B). The AUC value for lncRNA *SCAL1* was 0.61 (Sensitivity = 69%, Specificity = 60%, *P* = 0.0575) (Fig. 2 C). According to AUC values, *RMST* lncRNA had better performance in differentiating disease status in the study individuals compared with other genes.

**Fig. 3:**
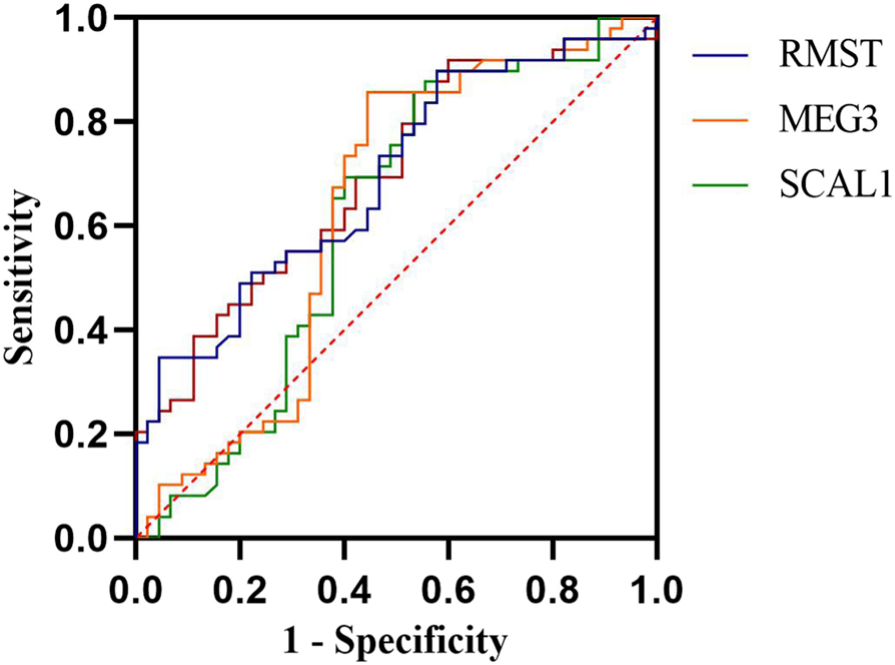
ROC curve for combination of expression levels of *RMST*, *MEG3*, and *SCAL1*

Figure 3 shows the ROC curve for combination of expression levels of *RMST*, *MEG3*, and *SCAL1* (AUC = 0.70, Sensitivity = 70%, Specificity = 61%, *P* = 0.001)*.*

## Discussion

The exact mechanism of BD is unclear. BD is a multifactorial disease, and various factors such as genetics and environmental parameters are involved in its etiology. There is strong evidence that oxidative stress partakes in the pathogenesis of the BD [28]. Also, some lncRNAs play an essential role in regulating oxidative stress [29]. As a result, in this research project, we examined the expression of five lncRNAs involved in the oxidative stress pathway in healthy and BD patients. Our study showed that expression levels of lncRNAs *MEG3*, *RMST* and *SCAL1* were down-regulated in cases, but no significant difference was shown in expression levels of lncRNAs *H19* and *MT1DP* between controls and patients. Similar results were observed in men. However, there was no significant difference in expression of lncRNAs between female BD cases and healthy women except for the *RMST* lncRNA.

*MEG3* is a maternally imprinted gene being expressed in many normal tissues. This lncRNA is involved in the differentiation of GABAergic neurons [30]. Interestingly, up-regulation of *MEG3* expression increases proinflammatory cytokines, decreases oxidative stress and apoptosis, and increases hippocampal neurons survival by activating the PI3K/AKT/mTOR pathway [31]. Meanwhile, this gene mediates ischemic nerve death by activating p53 and apoptosis [32]. Decreased expression of *MEG3* lncRNA has been reported in schizophrenia, Huntington’s disease and epilepsy [33]. Consistent with these studies, decreased expression of *MEG3* lncRNA in the present study may lead to increasing oxidative stress and reducing hippocampal neurons survival by inactivating the PI3K/AKT/mTOR pathway. Consequently, *MEG3* might act as a regulatory factor in the pathogenesis of BD through this axis.

*SCAL1* is located on chromosome 5 and is involved in tumorigenesis. Bhattacharjee et al. have shown that decreased SCAL1 gene expression was associated with inhibition of cell proliferation and increased apoptosis through decreasing expression levels of Bcl-2, while increasing Bax, Bad, and caspase-3 [34]. Studies have shown that *SCAL1* is strictly related to *Nrf2*, and decreased expression of *SCAL1* or *Nrf2* significantly increases cigarette smoke toxicity [34]. In addition, downregulated expression of *SCAL1* inhibits Nrf2 and cellular activity and induces oxidative stress via the Nrf2/Keap1 pathway [17]. Although the mechanism of *SCAL1* partake in BD is not known, we suggest that decreased expression of lncRNA *SCAL1* might affect the etiology of this disorder through enhancing oxidative stress and activating the Nrf2/Keap1 pathway.

The *RMST* gene is highly expressed in dopaminergic neurons in the midbrain [35]. Recent studies have shown that the *RMST* gene is controlled by REST and is involved in the differentiation of neurons in collaboration with the transcription factor SOX2 [19, 36]. *RMST* indirectly activates the p53/miR-107 signaling through interaction with the heterogeneous nuclear protein ribonucleus K (hnRNPK), which leads to an increase in BCL2 and eventually neuronal apoptosis [37]. Decreased expression of *RMST* lncRNA is also associated with increased oxidative stress. Silencing *RMST* expression reduces the size of the infarction and improves the results of the neural function test. Also, a decrease in markers of cerebral microgliosis and astrocytosis was also observed in the hippocampal region [38]. Considering the decreased lncRNA *RMST* expression in our study and previous information about this gene, such as its high expression in the brain, its role in neuronal differentiation, and oxidative stress regulation, this gene may be involved in the neurobiology of BD.

*H19* is involved in neuronal differentiation [39], and epigenetic variation in ICR Igf2/*H19* is associated with cerebellar development [40]. Research has shown that increasing the expression of *H19* lncRNA increases Bax and caspase-3 expression and decreases levels of Bcl-2, and induces apoptosis of hippocampal neurons by suppressing the expression of let-7b microRNA [41]. Also, up-regulation of expression of *H19* induces oxidative stress [12]. This gene is related with the initiation and prognosis of ischemic stroke [42]. In addition, expression of this lncRNA is increased in schizophrenia compared to healthy individuals. *H19* might partake in the pathogenesis of psychiatric disorders by increasing neuronal apoptosis.

*MT1DP* is a pseudo-gene from the metallothionein (MT) family. Gao et al. have shown that *MT1DP* stimulates cadmium-induced oxidative stress by suppressing Nrf2 and increasing miR-365 expression. Increased expression of *MT1DP* lncRNA leads to decreased cell proliferation and increased apoptosis [41]. Also, this gene interacts with the RhoC protein. Following cadmium-associated stress, the *MT1DP*/RhoC complex rapidly activates the RhoC-CCN1/2-AKT signaling. It enhances the flow of Ca2 + into the cell, leading to increased cadmium uptake and toxicity, and eventually results in cell death. MT1H, meanwhile, binds to miR-214 as a competitive endogenous RNA (ceRNA) to prevent suppression of *MT1DP* [43, 44].

We demonstrated significant differences of the expression levels of lncRNAs *MEG3* and *SCAL1* only between male BD patients and male controls, which might show distinctive roles of these lncRNAs among males and females. There are a bulk of evidence suggesting a potential differential expression of lncRNAs in BD and other neuropsychiatric disorders based on the sex. Firstly, a study in post-mortem brain tissues has shown dysregulation of *XIST* in female BD cases [45]. Moreover, cortical transcriptome analyses have shown sex-biased pathways in BD [46]. Sexually dimorphic transcriptomic networks have also been identified in BD cases, particularly in the cholinergic system [47]. A former study has shown sex-specific dysregulation of *HOXA-AS2*, *Linc-ROR*, *MEG3*, *SPRY4-IT1* and *UCA1* lncRNAs in patients with schizophrenia [48].

In the period of study, we recruited all patients with the mentioned criteria. This resulted in the male/female ratio of 70%/30% in cases. Then, we recruited the controls with this male/female ratio. Thus, an alternative explanation for lack of significance among female subgroups might be the smaller sample size of these subjects.

In addition, we displayed several pairwise correlations between expressions of lncRNAs in patients with BD suggesting similar functional roles in same signaling pathways and processes and their regulation by similar epigenetic mechanisms.

We also reported that *RMST* and *MEG3* may be useful for differentiating disease status in the study individuals. However, larger sample size is required for verifying these results and supporting the role of lncRNAs as diagnostic biomarkers in BD. In fact, we state small sample size as a limitation of our study.

## Conclusion

As patients have been under treatment with Carbamazepine, one might speculate that differences in expression of genes might be due to the effects of medication. However, if all expression differences were due to medication effects, analysis of expression of these genes might help in the assessment of patients’ adherence to administered treatments or even risk of disease-related complications.

In brief, our study demonstrated that the expression levels of lncRNA *MEG3* and lncRNA *RMST* were down-regulated in peripheral blood of BD patients.

## Data Availability

The datasets used and/or analyzed during the current study are available from the corresponding author on reasonable request.

## Abbreviations

lncRNAs: Long non-coding RNAs
BD: Bipolar disorder
ROC: Receiver Operating Characteristic
MT: Metallothionein
ceRNA: Competitive endogenous RNA
